# Serum Carcinoembryonic Antigen-Related Cell Adhesion Molecule 1 Level in Patients with Hepatocellular Carcinoma

**DOI:** 10.31557/APJCP.2021.22.11.3521

**Published:** 2021-11

**Authors:** Ekawee Sripariwuth, Setthachai Piwchan, Sutatip Pongcharoen

**Affiliations:** 1 *Division of Gastroenterology, Department of Medicine, Faculty of Medicine, Naresuan University, Phitsanulok, Thailand. *; 2 *Division of Immunology, Department of Medicine, Faculty of Medicine, Naresuan University, Phitsanulok, Thailand. *

**Keywords:** CEACAM1, hepatocellular carcinoma, HCC, serum

## Abstract

**Objective:**

To evaluate the clinical value of carcinoembryonic-antigen-related cell-adhesion molecule 1 (CEACAM1) in predicting the severity of hepatocellular carcinoma(HCC).

**Methods:**

We evaluated 40 healthy subjects and 40 HCC patients by collecting venous blood for the comparison. Serum CEACAM1 was detected using the Human CEACAM1 ELISA Kit. Other laboratory chemistries were analyzed by standard methods.

**Results:**

The serum level of CEACAM1 was not different between HCC patients and healthy subjects (p=0.0069). There was a correlation between serum CEACAM1 level and total bilirubin, and direct bilirubin. There was also a statistically significant difference among serum CEACAM1 levels stratified by BCLC staging and MELD score at the cut-point of 18. Lower platelet count, higher levels of aspartate aminotransferase, alanine aminotransferase and alkaline phosphatase were observed in HCC patients.

**Conclusion:**

An increase of serum CEACAM1 level was associated with cholestasis. The role of this molecule in HCC diagnosis was unclear. However, serum CAECAM1 may be useful to predict the severity in HCC patients.

## Introduction

Hepatocellular carcinoma (HCC) is the most common primary liver cancer worldwide (Singal et al., 2020). Multiple risk factors are considered to contribute in the development of this cancer, such as hepatitis B/C infection, cirrhosis, diabetes and tobacco (El–Serag and Rudolph, 2007). The mortality rate of this cancer is still high despite highly effective therapeutic modalities (Villanueva and Llovet, 2011). 

The diagnostic criteria of HCC include the finding of a hypervascular mass in the liver imaging, elevation of alfa-fetoprotein level or the tissue diagnosis (Heimbach et al., 2018). There are some limitations in these criteria which are small tumor size, poor sensitivity of alfa-fetoprotein and false negative of tissue diagnosis. Many biologic markers are proposed to be valued in the early diagnosis and prognosis prediction of HCC (Behne and Copur, 2012). 

The carcinoembryonic-antigen-related cell-adhesion molecule1 (CEACAM1) is the glycoprotein that is expressed on the cell membrane of most immune cells (Gray-Owen and Blumberg, 2016). CEACAM1 can be measured in serum, bile, urine and saliva. It functions in metabolism and tissue homeostasis and may be used to predict the prognosis of various cancers, such as melanoma, colorectal cancer, non-small cell lung cancer, pancreatic cancer and bladder cancer (Dankner et al, 2017). 

There is a paucity of information about CEACAM1 and HCC. One study has shown that the down regulation of CEACAM1 expression determines the aggressive behavior and poor prognosis for patients with HCC (Cruz et al, 2005). A second study has demonstrated CEACAM1 with a long cytoplasmic domain (CEACAM1-L) may be associated with the HCC invasiveness (Kiriyama et al, 2014). A third study has indicated the expression of CEACAM1 in the HCC tissue (Mao et al., 2017).

The authors hypothesized that the serum level of CEACAM1 in HCC patients may be different from that of healthy subjects and would be a potential biomarker for diagnosis and correlated with the prognosis compared with healthy subjects.

## Materials and Methods

This present work was a cross sectional single-center 

study performed at Naresuan University Hospital, Phitsanulok, Thailand.

Healthy subjects in the health check-up clinic and HCC patients in the HCC clinic over 20 years of age were enrolled from April 2020 to July 2020. The AASLD 2018 criteria were used for the HCC diagnosis (Heimbach et al., 2018). The exclusion criteria were the presence of another tumor.

The demographic data, cause of cirrhosis, MELD score and Barcelona Clinic Liver Cancer (BCLC) staging were collected. Venous blood was drawn for CECAM1 analysis. Serum CEACAM1 was detected using the Human CEACAM1 ELISA Kit ab215540 (Abcam, Cambridge, UK) according to the manufacturer’s instruction. The antibody in this kit was raised against the full extracellular domain of CEACAM1. This quantitated the serum CEACAM1 with 9.1 pg/ml sensitivity. The absorbance was read at 450 nm using the iMark Microplate Reader (Bio-Rad, Hercules, CA, USA).

The sample size was calculated by using the serum concentration of CEACAM1 in the previous study (Yang et al, 2015). The calculated sample size which aimed to meet a 95% confidence interval (alfa=0.01) was 80.

For baseline characteristics, numerical data are presented as descriptive statistics, mean with standard deviation or median with interquartile range, and tested for significant differences using independent t-test or the Wilcoxon Rank Sum (Mann-Whitney U) test as appropriate. Categorical data were concluded as proportion and percentage. For assessing statistical differences, Pearson Chi-square test or Fisher’s exact test was performed. Gaussian or Poisson regression model was used to identify the association between CEACAM1 level and hepatocellular carcinoma for multivariable analysis after considering significant factors, if the univariable analysis showed statistical significance. The distinction among serum CEACAM1 levels classified by HCC staging, according to BCLC staging, was also evaluated by using the Kruskal-Wallis test or one-way ANOVA test, as relevant. All p-values were represented as a two-sided hypothesis test, and the significant level for all tests was α less than 0.05.

## Results

The present study includes 80 patients divided into 40 HCC cases and 40 healthy subjects. The mean age of HCC and healthy subjects was 60±10.8 years and 43±10.7 years, respectively (p-value < 0.001). Of these, there were 32 (80%) males in the HCC group and 17 (42.5%) males in the healthy group (p-value = 0.001). Other baseline characteristics are presented in Table 1. HCC patients were older and had a lower platelet count. All of the HCC patients had cirrhosis and showed higher levels of aspartate aminotransferase, alanine aminotransferase and alkaline phosphatase compared with healthy subjects.

 The etiologies of cirrhosis were hepatitis B virus (HBV) infection (40%), hepatitis C virus (HCV) infection (35%), alcoholic liver disease (15%), metabolic-associated liver disease (5%), and HBV & HCV co-infection (5%).

The association between serum CEACAM1 level and hepatocellular carcinoma was evaluated for the univariable method, using the Wilcoxon Rank Sum (Mann-Whitney U) test due to the non-normal distribution of the data. The result showed no statistical association (p-value = 0.069) (Table 1).

There were no statistically significant differences in serum CEACAM1 level among various etiologies of cirrhosis (Table 2). The serum CEACAM1 levels in HCC patients with HBV or HCV infection were also not different (Table 3 and 4). On the contrary, the study revealed statistically significant differences among the serum CEACAM1 levels when stratified by the BCLC staging (Table 5) and MELD score at the cut-point of 18 (Table 6).

The correlation between serum total bilirubin (TB) and direct bilirubin (DB) level with serum CEACAM1 level in HCC patients was also calculated using Spearman’s rank correlation analysis owing to the non-normal distribution of the data (mean TB ±SD = 1.64±3.45 mg/dL, median TB, IQR: 0.81, 0.58-1.48 mg/dL and (mean DB±SD : 1.06±3.03 mg/dL, median TB, IQR: 0.39, 0.26-0.72 mg/dL). The analysis showed a positive correlation statistically significant between serum CEACAM1 level and serum total bilirubin, as well as serum direct bilirubin level. Spearman’s rank correlation demonstrated a Spearman rho of 0.32 for correlation between serum total bilirubin and CEACAM1 level, and a Spearman rho of 0.43 for the correlation between serum direct bilirubin and CEACAM1 level with a p-value of 0.047 and 0.006, respectively.

**Table 1:** Baseline Characteristics

Results	HCC Patients (N=40)	Healthy Subjects (N=40)	p-value
Mean age ± SD (Years)	60 ± 10.8	43 ± 10.7	<0.001
Male (N, %)	32, 80%	17, 42.5%	0.001
Mean hemoglobin ± SD (g/dL)	12.3 ± 2.1	13.3 ± 1.4	0.014
Mean white blood cells ± SD (cells/mm^3^)	6,699 ± 3,138	6,611 ± 1,275	0.871
Median platelet (x10^3^/mm^3^)	155.5 (113-179)	275.5 (243-325.5)	<0.001
Median aspartate aminotransferase (U/L)	73 (46-125)	21 (16-24)	<0.001
Median alanine aminotransferase (U/L)	40 (27-79)	16 (12-20)	<0.001
Median alkaline phosphatase (U/L)	111 (94-165)	58 (45-68)	<0.001
Mean blood urea nitrogen ± SD (mg/dL)	13.8 ± 6.7	12 ± 3.4	0.146
Mean creatinine ± SD (mg/dL)	1.01 ± 0.37	0.81 ± 0.18	0.004
Median of serum CEACAM1 level (pg/mL)	1,692.91 (1,125.68-2,412.80)	1,332.67 (1,070.59-1,707.85)	0.069

**Table 2:** Serum CEACAM1 Level Stratified by Etiologies of Cirrhosis

Etiology (N)	Mean ± SD	Median (IQR)
HBV (16)	2,059.86 ± 1,810.04	1,642.35 (1,020.93-2,433.34)
HCV (14)	1,861.22 ± 1,411.04	1,432.41 (918.55-2,105.08)
Alcohol (6)	2,081.25 ± 857.11	1,735.97 (1,592.52-2,929.99)
NASH (2)	1,918.82 ± 50.59	1,918.82 (1,883.05-1,954.59)
HBV + HCV (2)	2,418.8 ± 89.14	2,412.8 (2,349.77-2,475.83)

**Table 3:** Serum CEACAM1 Level in HCC Patients with and without HBV Infection

HBV (N)	Mean ± SD	Median (IQR)	p-value
Yes (18)	2,099.07 ± 1,704.20	1,718.82 (1,135.95-2,475.83)	0.957
No (22)	1,926.47 ± 1,190.49	1,678.95 (1,115.40-2,105.08)	

**Table 4:** Serum CEACAM1 Level in HCC Patients with and without HCV Infection

HCV (N)	Mean ± SD	Median (IQR)	p-value
Yes (16)	1930.17 ± 1,327.24	1,642.13 (944.57-2,412.8)	0.761
No (24)	2,053.45 ± 1,516.01	1,729.03 (1,153.28-2,433.34)	

**Table 5:** Serum CEACAM1 Level Stratified by BCLC Staging

BCLC stage	Mean ± SD	Median (IQR)	p-value
0 (N=1)	571.593	571.593	0.037
A (N=11)	1476.23 ± 952.95	1,159.74 (907.91-1,654.76)	
B (N=19)	2,266.12 ± 1,739.89	1,754.94 (1,235.32-2,732.90)	
C (N=7)	2,500.35 ± 991.50	2,475.83 (1,768.81-3,197.63)	
D (N=1)	662.923	662.923	

**Table 6:** Serum CEACAM1 Level Stratified by MELD Score

MELD	Mean ± SD	Median (IQR)	p-value
<18	1,694.90 ± 1,018.05	1,615.76 (1,115.40-1,954.59)	0.022
≥18	4,042.80 ± 2,637.15	3,380.46 (2,207.82-5,877.78)	

## Discussion

CEACAM1 (biliary glycoprotein or CD66) was first discovered in the bile of a patient with biliary obstruction. Its level correlated with cholestasis (Svenberg et al., 1981). In this study, the serum CEACAM1 level increased in HCC patients which may indicate a cholestatic condition in these patients. However, the elevation of serum CEACAM1 level was not significantly different when compared with healthy subjects. This could be explained by the number of patients with early stage HCC where cholestasis may not be severe enough to produce a high level of serum CEACAM1.

To date, there has been no report about soluble CEACAM1 and HCC. Our study revealed that the level of serum CEACAM1 correlated with the high BCLC stage and high MELD score. A prior study has shown the association between the loss of CEACAM1 expression in tumor tissue and aggressive tumor behavior (Cruz et al., 2005). The tissue CEACAM1 is expressed at the canalicular membrane. It needs to be absorbed into the systemic circulation before presenting in the serum. Hence, a low tissue CEACAM1 expression should result in a low serum CEACAM1 level. The discordant results between the two studies may indicate that the soluble CEACAM1 and the tissue CEACAM1 are different isoforms. The differential expression of CEAMCAM1 in tissues and serum might also reflect distinct features of pathophysiological tissues as well as of the host immune responses as the molecule is also produced by activated T cells (Beauchemin et al., 2013).

Hepatitis B infection may influence the expression of CEACAM1. One study suggested that only 50% of HBV-related HCC patients express CEACAM1 in HCC tissue (Mao et al., 2017). Almost half of the HCC patients had an HBV infection (40%). The tumor tissue in these patients may not express high enough CEACAM1 to produce a significant level of secreted CEACAM1 in the serum. 

A previous study has shown that the CEACAM1 expression is found in well-differentiated HCC (Tanaka et al, 1997). Unfortunately, there were no tissues for the diagnosis in our study. High levels of serum CEACAM1 in our study could be due to the degree of cholestasis or tissue type of the well-differentiated HCC as well.

There are some limitations in this study. The laboratory data in healthy subjects were not complete because it was determined by the program of annual health check-ups. The Child Turcotte Pugh score could not be calculated due to the incompleteness of laboratory results. Most of the HCC patients in our hospital were diagnosed by radiologic criteria. The HCC tissue was not available for data processing. The isoforms of CEACAM1 were not investigated. 

This study demonstrates the new knowledge that the serum CEACAM1 has potential to be the biomarker of HCC in terms of disease severity. It is more convenient to measure this molecule in the serum. However, the role of serum CEACAM1 and its isoform in HCC diagnosis, progression and prognosis is not completely understood. Future studies are needed to demonstrate the true benefit of this molecule.

## Author Contribution Statement

Ekawee Sripariwuth: study concept and design, acquisition of data, drafting of the manuscript critical revision of the manuscript for important intellectual content, funding, and administrative, technical, or material support and study supervision. Setthachai Piwchan: drafting of the manuscript and statistical analysis. Sutatip Pongcharoen: study concept and design, drafting of the manuscript and technical and material support.

## Funding Statement

Ekawee Sripariwuth received a research grant from the Faculty of Medicine, Naresuan University (MD2563C006). Sutatip Pongcharoen received funding from the National Science, Research and Innovation Fund (R2564B011, R2565B001) and Thailand Science, Research and Innovation (BRG6180010).

## Ethics approval

This study was approved by by the Naresuan University Institutional Review Board (IRB No.1061/61).

## Name the ethical committee

Naresuan University Institutional Review Board has approved this study. All subjects gave their written informed consent. The study protocol conformed to the ethical guidelines of the 1975 Helsinki Declaration and International Conference on Harmonization in Good Clinical Practice (ICH-CGP).

## Availability of data

Data are available upon request.

## Conflict of Interest

The authors have no conflicts of interest to declare.
